# *Gongjin-Dan* Enhances Hippocampal Memory in a Mouse Model of Scopolamine-Induced Amnesia

**DOI:** 10.1371/journal.pone.0159823

**Published:** 2016-08-02

**Authors:** Jin-Seok Lee, Sung-Shin Hong, Hyeong-Geug Kim, Hye-Won Lee, Won-Yong Kim, Sam-Keun Lee, Chang-Gue Son

**Affiliations:** 1 Liver and Immunology Research Center, Oriental Medical Collage of Daejeon University, 22–5 Daehung-dong, Jung-gu, Daejeon, 301–724, Republic of Korea; 2 Korean Medical College of Daejeon University, 22–5 Yongwoon-dong, Dong-gu, Daejeon301-724, Republic of Korea; 3 TKM-based Herbal Drug Research Group, Korea Institute of Oriental Medicine, Daejeon 305–811, Republic of Korea; 4 Department of Applied Chemistry, Daejeon University, 62, Daehak-ro, Dong-gu, Daejeon 34520, Republic of Korea; UPMC, FRANCE

## Abstract

We evaluated the neuropharmacological effects of *Gongjin-Dan* (GJD) on the memory impairment caused by scopolamine injection. BALB/c mice were orally treated with GJD (100, 200, or 400 mg/kg, daily) or tacrine (THA, 10 mg/kg) for 10 days, and scopolamine (2 mg/kg) was injected intraperitoneally. The radial arm maze and passive avoidance tests were performed to evaluate the animal’s learning and memory. Scopolamine increased the task completing time, the number of total errors (reference and working memory error) in the radial arm maze task, and the latency time in the passive avoidance test, which were significantly ameliorated by treatment with GJD. The GJD treatment also attenuated the scopolamine-induced hyperactivation of acetylcholinesterase activity, and suppression of the expression of brain-derived neurotrophic factor (BDNF), nerve growth factor (NGF) and their receptors in the hippocampus. These effects of GJD were supported by both the doublecortin (DCX)-positive staining and Nissl staining, which were used to measure hippocampal neurogenesis and atrophy, respectively. These findings strongly suggest that GJD exerts a potent anti-amnesic effect, and its underlying mechanism might involve the modulation of cholinergic activity.

## Introduction

As the population ages, the burden of neurodegenerative disorders becomes a globally critical issue. The number of patients suffering from Alzheimer’s disease has risen rapidly; for example, in 2015 approximately 46.8 million people worldwide were diagnosed with this disease [1]. Among the signs or symptoms of neurodegenerative disorders, memory impairment is the most typical feature, which results from neuronal dysfunction and neuronal loss in the brain tissues, particularly the hippocampal region [2].

The pathogenesis of neurodegenerative disorders still remains uncertain; however, the deposition of neurofibrillary tangles and/or senile plaques, neuroexcitotoxicity, and cholinergic dysfunction are known to be causative factors [3]. The cholinergic system, including cholinergic neurons, neurotransmitters and their specific receptors plays a central role in the memory process (encoding, memory storage and retrieval) [4]. As a neurotransmitter, acetylcholine obviously enhances long-term potentiation (LTP) in the basal forebrain and hippocampus [5]. These cholinergic functions are mediated by neurotrophins such as brain-derived neurotrophic factor (BDNF) and nerve growth factor (NGF), which maintain neuronal plasticity and synaptogenesis [6].

Therefore, cholinergic abnormalities are closely linked to neurodegenerative disorders [7]. Acetylcholinesterase inhibitors and the agents that modulate cholinergic dysfunction, such as donepezil, rivastigmine, galantamine and tacrine, have been used to treat patients with amnesia-related disorders such as Alzheimer’s disease [8]. However, these agents are not curatives, but are used to delay the progression of disease and ameliorate the pathognomonic symptoms [9]. These drugs were also observed to have severe adverse effects such as hepatocytotoxicity, vomiting and nausea [10]. Meanwhile, several study groups have recently analyzed the memory enhancing activities of herb-derived natural products [11, 12].

*Gongjin-Dan* (GJD) is a commercially available remedy in Asia, consisting of herbs and animal-derived materials, which have been traditionally prescribed to patients with weak constitution and to the aged population based on a traditional medical text called *Dongui-Bogam* [13]. GJD is currently prescribed for patients with central fatigue disorder or central nervous system diseases [14]. Previous studies suggested that GJD has a memory enhancing activity and neuroprotective effect on the animal models of ischemic stroke [14, 15]. Moreover, the anti-chronic fatigue effect of GJD was shown in our previous study [16]. These facts suggested that GJD could ameliorate cholinergic dysfunction and could be a candidate anti-amnesic drug.

In this study, we adopted a mouse model of scopolamine-induced memory deficits to investigate the pharmacological actions of GJD on memory impairment and its underlying mechanisms.

## Materials and Methods

### Preparation

*Gongjin-Dan* (GJD, 329-H340753) was purchased from the Kyung-Bang pharmacy (Incheon, Korea). The manufacturer confirms that each ingredient in every batch met the quality control guidelines of the Ministry of Food and Drug Safety (MFDS). GJD is composed of three medicinal herbs (*Corni fructus*, *Korea angelica*, *and Ginseng radix*) and two animal-derived materials (*Muschus* and *Cornus cervi parvum*). The ground ingredients are formed into a pill, which is enclosed with gold leaf (3.75 g, total weight/pill). The detailed information about GJD is summarized in Table 1.

**Table 1:** Composition of *Gongjin-Dan*.

Herbal name		Scientific name (Local name)	Reference compound	Quantity (mg/pill)	Amount (mg/pill)
**Herbs**	Corni fructus	*Cornus officinalis* Siebold et Zucc.	Loganin	2.67± 0.24	444.4 (11.85%)
	Korea angelica	*Angelica gigas Nakai*	Decursin	29.71± 3.01	444.4 (11.85%)
	Ginseng radix	*Panax ginseng* C.A. Meyer	Ginsenoside Rb1	0.91± 0.07	444.4 (11.85%)
**Animal derives**	Muschus	*Moschus moschiferus L*.	*L*-muscone	1.48± 0.11	74.0 (1.97%)
	Cornus cervi parvum	*Cervus nippon Temminck*	Ganglioside	6.35± 0.52	444.4 (11.85%)
**Diluting agents**	Mel	*A*. *indica* Radoszkowski			1,898.4 (50.63%)
	Aurum	Gold			Quality standard
Total:					3,750 (100%)

According to the therapeutic dose of one pill per 60-kg adult daily, the GJD dose for this study was a maximum of 400 mg/kg per day per mouse. GJD was dissolved in distilled water, and it was used to animal study.

### Fingerprinting and quantification of GJD

**Fig 1:**
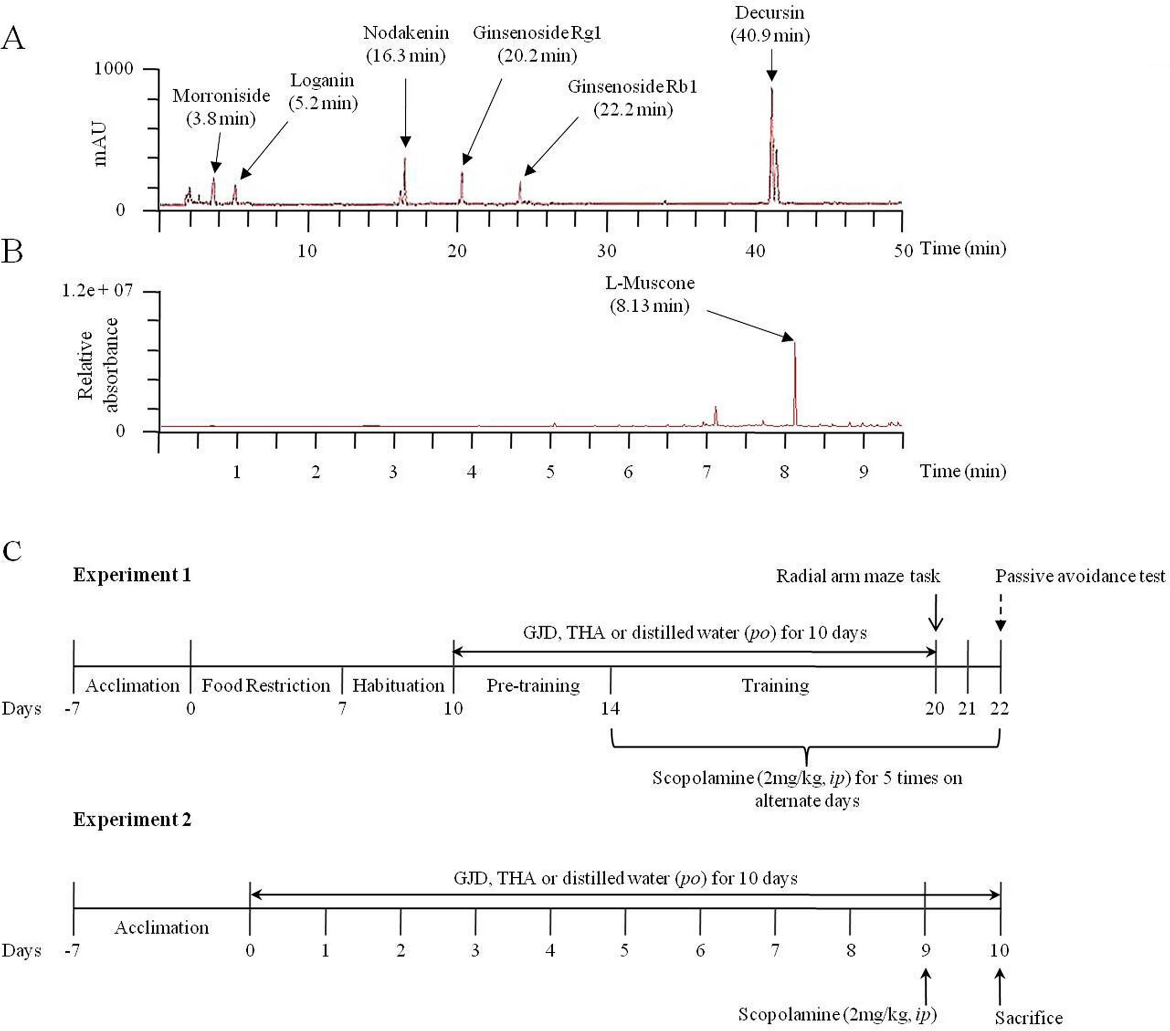
Fingerprint of GJD and experimental scheme. The GJD and its 6 main components were subjected to HPLC. All chromatograms were obtained at a wavelengths of 203 Ginsenoside Rg1 and Rb1), 240 (morroniside and loganin), and 330 nm (nodakenin and decrusin). The detected peaks were merged (A). The L-muscone in Muschus was detected by gas chromatography (B). Two experimental schemes were shown (C).

For the fingerprinting analysis of GJD and its active compounds, the GJD and the reference compounds were dissolved in absolute methanol. The reference compounds for each ingredient (the ginsenoside Rb1 and Rg1 for *Ginseng radix*, morronoside and loganin for *Cornus cervi parvum* and nodakenin and decrusin for *Korea angelica*) were submitted to high-performance liquid chromatography (HPLC). All of the calibration curves were obtained by assessing the peak areas for the six concentrations of each compound, ranging from 15.63 to 500 μg/mL. The linearity of the peak area (y) versus concentration (x, μg/mL) curve for each component was used to calculate the contents of the main components of GJD. The quantitative analysis was simultaneously performed using an 1100 series HPLC (Agilent Technologies, Santa Clara, CA) equipped with an autosampler (G11313A), column oven (GA1316A), binary pump (G1312), diode-array-detector, and degasser (GA1379A). The analytical column was a Gemini C18 column (4.6×250 mm; particle size 5 μm; Phenomenex, Torrance, CA), and quantitative data was acquired by the Chemstation software. The mobile phases contain 10% acetonitrile (with 0.05% formic acid) and 90% acetonitrile in water. The injection volume was 10 μL, and gradient flow was as follows; 0–15 min, 0–15% B; 15–20 min, 35–65% B; 20–45 min, 65–35%; 45–60 min, 100–0%, respectively. The HPLC was operated at a flow rate of 1.0 mL/min, and the wavelengths monitored were 203, 240, and 330 nm (Fig 1A).

For the fingerprinting analysis of L-muscone in GJD, GJD was extracted with ethyl acetate that had been distilled from potassium carbonate. The extract was centrifuged and ultrasonicated for 15 min, respectively. The supernatant was concentrated under reduced pressure at 15°C. Agilent 7890A gas chromatograph/5975C mass selective detector (GC/MSD) equipped with Agilent G1701EA GC/MSD Chemstation software was used. The GC was equipped with an Agilent J&W HP-5ms inert GC column (30 m length; 2.25 mm ID; 0.25 μm film thickness). The injector and interface temperatures were maintained at 260 and 280°C, respectively. The oven temperature was held at 100°C for 1 min, programmed to 280°C at 20°C/min, and then held at the final temperature for 10 min. The following parameters were used when injecting the sample into the GC-MSD system: a sample size of 1 μL using the injection mode with a split ratio of 10:1. The full-scan mass spectrometric data were acquired for the m/z 50–550 range. The selective ion monitoring (SIM) data were collected with m/z 238, 223, and 209 peaks denoting the presence of L-muscone. The quantification of muscone in GJD was calculated using five standard solutions (0.6, 1.0, 1.2, 2.0, and 2.4 μg/mL of muscone). The concentrations were expressed in μg/mL and then converted into μg/mg and % by taking the volume of the extract (1.3 mL) and the sample size (3.75 g for GJD) into consideration.

### Chemicals

The following reagents were obtained from Sigma (St. Louis, MO, USA); acetic acid, bicinchoninic acid solution, bovine serum albumin, copper(II) sulfate pentahydrate, Cresyl violet acetate, 3,3′-diamino-benzidine (DAB), radioimmunoprecipitation assay (RIPA), scopolamine hydrobromide, skim milk, and 9-amino-1,2,3,4-tetrahydroacridine hydrochloride hydrate (THA). The CREB, phospho-CREB, NGF, BDNF, beta-actin, and secondary horseradish peroxidase (HRP)-conjugated antibodies used for western blotting were obtained from Abcam (Cambridge, MA) and Santa Cruz Biotechnology (Santa Cruz, CA); the doublecortin (DCX), biotinylated secondary antibodies, and avidin-biotin peroxidase complex used for immunohistochemical staining were obtained from Abcam (Cambridge, MA), Santa Cruz Biotechnology (Santa Cruz, CA) and Vector Laboratories (Burlingame, CA).

### Cell culture

HT22 mouse hippocampal neuronal cells were kindly provided by Prof. Dong-Woon Kim (Chungnam National University, Daejeon, Korea). The cells were cultured in DMEM (Welgene) supplemented with 10% heat-inactivated FBS (Welgene) and 1% penicillin-strepto-mycin-amphotericin antibiotics (Welgene) at 37°C, with 5% CO_2_ humidified atmosphere.

### Animals

One-hundred twenty specific pathogen-free BALB/C male mice (10 weeks old; 24–26 g) were purchased from Dae-Han Biolink Company (Chungcheongbuk-do, Korea). The mice had access to water and food pellets (Cargill Agri Furina, Gyeonggido, Korea) *ad libitum*, and were housed in a room maintained at 23 ± 2°C with a 12-h: 12-h light-dark cycle. The protocols were approved by the Institutional Animal Care and Use Committee of Daejeon University (DJUARB2015-051) and were conducted in accordance with the Guide for the Care and Use of Laboratory Animals published by the U.S. National Institutes of Health.

After acclimatization for 1 week, the mice were randomly allocated into two experiments; experiment 1 was the memory-related behavioral test (n = 60) and experiment 2 was the biochemical analysis (n = 60). In each experiment, the mice (n = 60) were then divided into the following six groups (n = 10): vehicle, control, GJD treatment (100, 200 and 400 mg/kg), and positive control (10 mg/kg THA). GJD and THA were dissolved in distilled water and administered to each group by gavage daily for 10 days (for both experiments 1 and 2). In experiment 1, scopolamine (2 mg/kg, dissolved in 0.9% physiological saline) was intraperitoneally injected into the mice on alternate days (total five times between day 14 and day 22) after the pre-training periods (from experimental day 10 to day 14) on the radial arm maze task. In experiment 2, scopolamine (2 mg/kg) was intraperitoneally injected into the mice 24 h before sacrifice. The details of the experimental design are shown in Fig 1C.

### Radial arm maze task

The radial arm maze task was performed in a central octagonal platform (12 cm diameter) with eight arms (24 × 4 cm). The apparatus was elevated 45 cm above the floor, and it was located in a room with extra visual cues.

Before training task, mice were restricted food intake for 7 days to reduce their initial body weight by 10%. During this period, all mice were habituated to handing, weighing and apparatus of maze daily. For habituation, mice were freely allowed to explore the maze for 10 min, which was repeated twice a day for 3 days (at 1 h interval). Cereal rewards were randomly placed at the end of four arms, and its layout was kept during the performance of radial arm maze task. Next, mice were pre-trained on the radial arm maze for 4 consecutive days (three trials per day at 1 h interval). During the pre-training session, mice were placed in the central octagonal platform for 10 sec, and then allowed freely to eat four cereal rewards until they finished cereal (maximally for 5 min). The training was performed for 6 consecutive days (once daily) as same as pre-training. On the training day 7 (experimental day 20), the mice were allowed to finished reward cereal without time limitation. For 7 days testing, spatial memory were recorded using an EthoVision XT9 video tracking system and software (Noldus, Wageningen, Netherlands) as follows; 1) working memory errors (entries into already baited arms), 2) reference memory errors (entries and re-entries into non-baited arms), and 3) time to finishing cereal (only on day 7). Above protocol had been practiced after our pre-test confirmed that all mice had reached our criterion level (average working and reference memory error ≤ 1) on day 10 during the training session, and 2mg/kg of scopolamine injection (4 alternative days) doubled the memory error frequency comparing to vehicle group.

### Passive avoidance test

The passive avoidance test was performed in a two identical light-dark square boxes (12 × 10 × 12 cm, respectively) with a vertical sliding door (5 × 5 cm) between the boxes. The light box was illuminated by a bare 50 W bulb. The mice were initially placed in the light box for 10 sec, and then the door was opened to allow them to enter the dark box because mice instinctively prefer darkness. If a mouse entered the dark box, the door was closed and an electronic shock (0.5 mA) was administered to the mouse for 5 sec. After 24 h, the mice were again placed in the light box with the door opened. The latency to enter into the dark box was recorded for 5 min.

### Sample preparation

In experiment 2, all mice were sacrificed under ether anesthesia. The serum was collected by centrifugation at 3000 × *g* for 15 min at 4°C, and the hippocampi were immediately isolated. The sera and hippocampi were stored at -80°C or in RNAlater (Ambion, TX, USA). Three mouse brains from each group were fixed in 4% paraformaldehyde, and the hippocampus of remaining seven mice was divided to two parts. Part of the hippocampus was homogenized on ice using RIPA buffer, and the other part of hippocampus was used to isolate the RNA.

### AChE activity

The acetylcholinesterase (AChE) activity in the hippocampus was determined using an AChE activity assay kit (AAT Bioquest; Sunnyvale, CA, USA) according to the manufacturer’s protocol. The absorbance at 410 nm was measured using a UV spectrophotometer.

### Western blot analysis

The expression of the CREB/phospho-CREB, BDNF, and NGF proteins in the hippocampus was evaluated by Western blotting. The proteins were separated by 10% polyacrylamide gel electrophoresis and transferred to polyvinylidene fluoride (PVDF) membranes. After blocking in 5% skim milk, the membranes were probed with primary antibodies (CREB, pCREB, BDNF, NGF and β-actin) overnight at 4°C. The membranes were washed and incubated with an HRP-conjugated anti-rabbit antibody for 2 h. Western blots were visualized using an advanced enhanced chemiluminescence advanced kit.

The HT22 cells were seeded onto 60-mm dishes at a density of 2×10^5^ cells/dish. After treatment with GJD (0, 0.01, 0.1, 1, and 10 μg/mL) for 12 h, the cells were rinsed three times with PBS, and then cells were lysed with RIPA buffer. The lysates were centrifuged at 12,000 × *g* for 20 min at 4°C and the supernatants were quantified for the total protein using a bicinchoninic acid protein assay. The BDNF protein level of lysates was evaluated by western blotting.

### Quantitative real-time PCR analysis

The mRNA expression levels of the genes encoding mAChR1, TrkA, TrkB and synaptophysin in the hippocampus were measured by real-time PCR. The total RNA was isolated from the hippocampus using an RNeasy Mini Kit (QIAGEN, Valencia, CA, USA) and the cDNA was synthesized using a High-Capacity cDNA Reverse Transcription Kit (Ambion, Austin, TX, USA). Real-time PCR was performed using the SYBRGreen PCR Master Mix (Applied Biosystems; Foster City, CA, USA) and PCR amplification was performed using a standard protocol with the IQ5 PCR Thermal Cycler (Bio-Rad, Hercules, CA, USA). The sequences of the primers are listed in Table 2.

**Table 2:** Sequence of the primers used in real-time PCR analysis.

Gene (number)	Primer sequencing (Forward and Reverse)	Product size (base pair)	Annealing temperature (°C)
mAChR 1 (NM_001112697)	5'-AGT GGC ATT CAT CGG GAT CA-3'	100	60
	5'-CTT GAG CTC TGT GTT GAC CTT GA-3’		
TrkA (NM_001033124)	5'-TCAATCAGCCCACGCATGT-3'	100	62
	5'-TTGTCCATAAAAGCAGCCATGA-3’		
TrkB (NM_001025074)	5′-CCTGCGGCACATAAATTTCA-3′	104	58
	5′-CGGATTACCCGTCAGGATCA-3′		
Synaptophysin (NM_0099305)	5′-CCGCCAGACAGGAAACACAT-3′	100	60
	5′-AACCCAGAGCACCAGGTTCA-3′		
*β*-actin (NM_007393)	5'-GGC ACC ACA CCT TCT ACA ATG A-3'	100	59
	5'-ATC TTT TCA CGG TTG GCC TTA G-3'		

mAChR; muscarinic acetylcholine receptor, Trk; tyrosine receptor kinase, *β*-actin as a housekeeping.

### Histological and Immunohistochemical analysis

The brains were methodically cryoprotected in the 10, 20 and 30% sucrose for 24 h. These brains were embedded in the tissue-freezing medium (Leica Microsystems, Bensheim, Germany), frozen in liquid nitrogen, and cut into coronal frozen sections (35 μm) using a Leica CM3050 cryostat. The sections were stored in anti-freeze buffer.

These free-floating sections were mounted on glass slides, air-dried, and fixed by immersion in cold acetone (-20°C) for 2 min. To perform the Nissl staining, the slides were stained with 0.5% cresyl violet acetate for 20 min. Other free-floating sections were subjected to endogenous peroxidase quenching with 1% H_2_O_2_ in PBS, followed by treatment with blocking buffer (5% normal chicken serum in PBS and 0.3% Triton X-100 overnight at 4°C) and incubated with primary DCX (1:200, sc-8066, Santa Cruz Biotechnology) antibodies. After washing with PBS, the tissues were incubated with a biotinylated universal pan-specific antibody (1:200, BA-1300, Vector Laboratories). The tissues were subsequently exposed to an avidin-biotin peroxidase complex (Vectastain ABC kit, Vector Laboratories) for 2 h. The peroxidase activity was visualized using a stable diaminobenzidine solution. The Nissl straining and immunoreactions were observed using an Axio-phot microscope (Carl Zeiss, Germany) and these results were quantified using the Image J 1.46 software (NIH, Bethesda, MD, USA).

### Statistical analysis

All data are expressed as the means ± standard deviations (SD). The statistical significance of the differences between the groups were evaluated by one-way analysis of variance (ANOVA) followed by multiple *post hoc* multiple comparisons with Tukey’s HSD t-test using the IBM SPSS statistics software, ver. 20.0 (SPSS Inc., Chicago, IL, USA). Differences at *P* < 0.05, *P* < 0.01, or *P* < 0.001 were considered statistically significant.

## Results

### Fingerprinting analysis of GJD

The fingerprinting analysis of GJD was performed using HPLC and GC/MSD. Morroniside and loganin from *Cornus cervi parvum* were detected at retention time of 3.8 and 5.2 min, respectively. The retention times of nodakenin and decrusin from *Korea angelica* were 16.3 and 40.9 min, respectively. The ginsenoside Rg1 and Rb1 from *Ginseng radix* were detected at 20.2 and 22.2 min, respectively. The absorbances were confirmed at the 240 nm (morroniside and loganin), 330 nm (nodakenin and decrusin) and 203 nm (ginsenoside Rg1 and Rb1). These peaks with respect to each compound were merged in Fig 1A.

The L-muscone from the *Muschus* was analyzed by GC/MSD, and it was detected at a retention time of 8.13 min (Fig 1B). All of the components in GJD were subjected to a quantitative analysis, and they corresponded to the MFDS standards (Table 1).

### Anti-amnesic effects of GJD in the radial arm maze task

**Fig 2:**
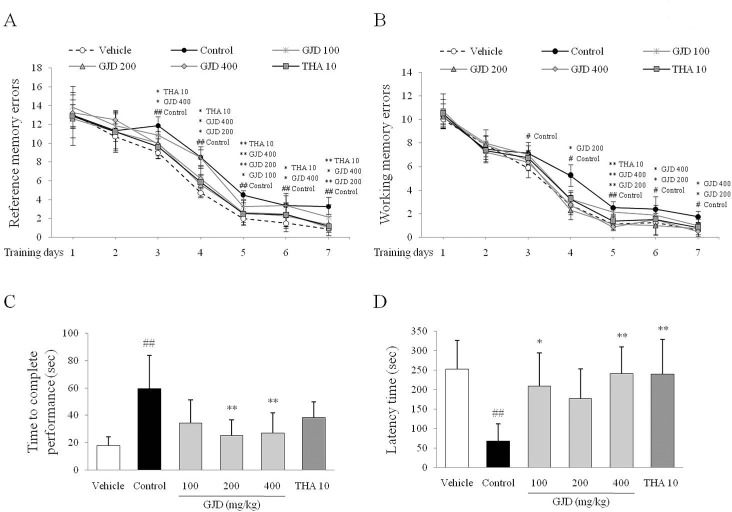
Behavioral tests for the memory. In the radial arm maze task, reference memory errors (A) and working memory errors (B) were recorded for 7 days (day 14–20 of the experiment). Time to complete the task (C) was recorded on experimental day 20. In the passive avoidance test, latency time was recorded on experimental day 22. The data are expressed as the means ± SD (*n* = 10). ^#^P < 0.05, ^##^P < 0.01, ^###^P < 0.001 compared with the vehicle group; *P < 0.05, **P < 0.01, and ***P < 0.001 compared with the control group.

When the reference and working memory errors were separately analyzed for 6 day of training period (day 14–19 of the experiment), the GJD treatment significantly enhanced the learning abilities compared with the control group (P < 0.05 or P < 0.01 or P < 0.001). Especially on the experimental day 20, the scopolamine injection significantly increased both reference and working memory errors compared with the vehicle group [F (5, 54) = 3.236; P < 0.01 and F (5, 54) = 5.782; P < 0.05, respectively], but both the working memory errors (P < 0.05 or P < 0.01) and reference memory errors (P < 0.05) were significantly ameliorated by the GJD treatment (200 and 400 mg/kg; Fig 2A and 2B). Besides, the scopolamine injection significantly increased the time to complete the task compared with the vehicle group [approximately 3-fold for both, F (5, 54) = 6.536; P < 0.01]. These amnesic behaviors were significantly attenuated by the GJD treatment compared with the control group (P < 0.01 for both 200 and 400 mg/kg; Fig 2C). THA significantly reduced the total number of entry error and reference memory errors.

### Anti-amnesic effects of GJD in the passive avoidance test

The scopolamine injection resulted in a significantly shorter latency time compared with the vehicle group [F (5, 54) = 5.279; P < 0.01]. The GJD treatment significantly increased the latency time compared with the control group (P < 0.05 for 100 mg/kg, P < 0.01 for 400 mg/kg; Fig 2D). THA had effects similar to the GJD treatment.

### Effects on the AChE activity in the hippocampus

**Fig 3:**
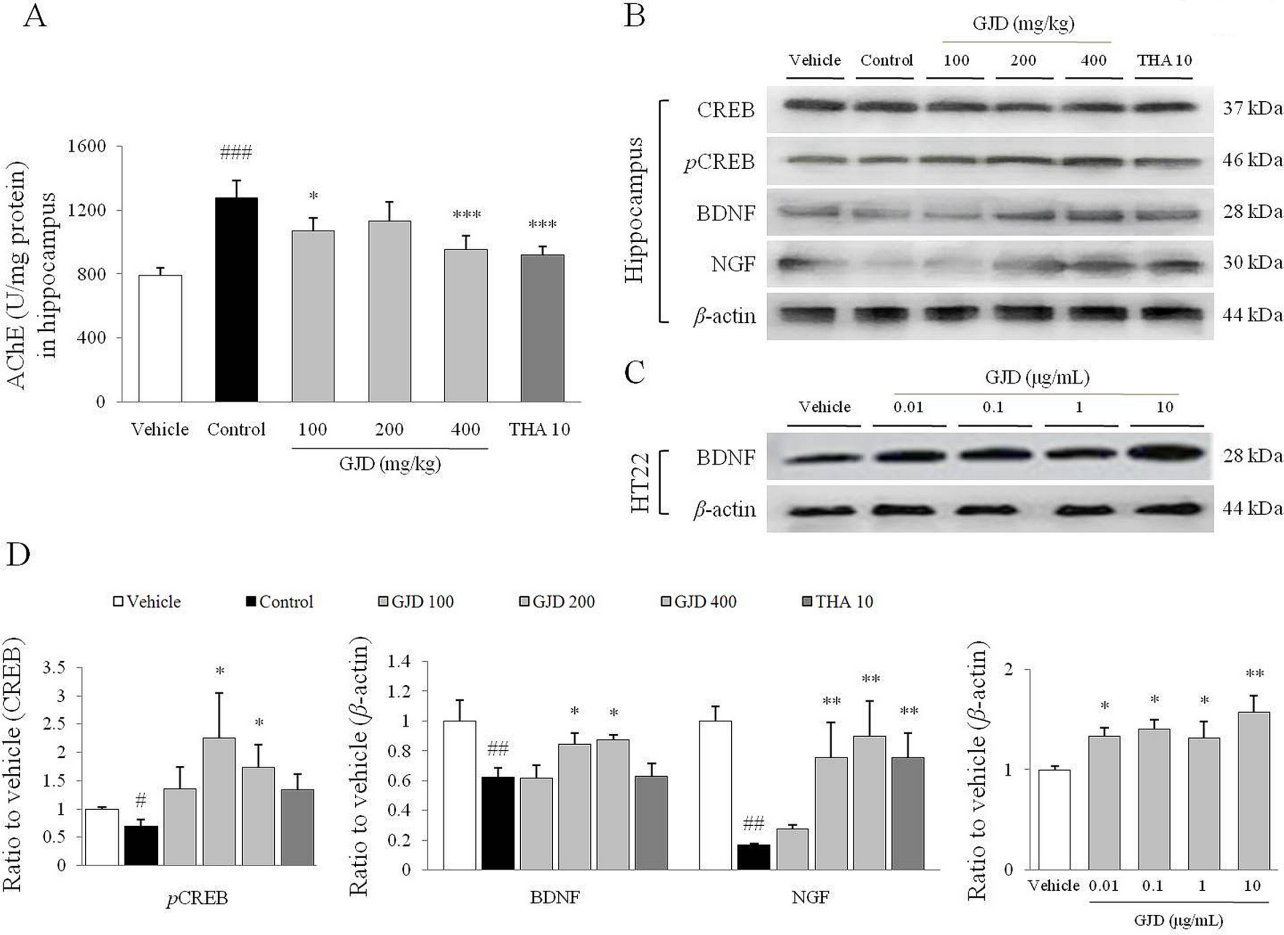
Hippocampal levels of the AChE, CREB and neurotrophic proteins. AChE activity (A) in the hippocampus was measured with an ELISA kit. The western blotting assay for the phosphorylated CREB, BDNF, and NGF levels in hippocampal tissues (B) and BDNF levels in HT22 cell (C), and their relative intensities (D) were shown. The data are expressed as the means ± SD (*n* = 7 or 3). ^#^P < 0.05, ^##^P < 0.01, and ^###^P < 0.001 compared with the vehicle group; *P < 0.05, **P < 0.01, and ***P < 0.001 compared with the control group.

The AChE activity in the hippocampus was significantly increased by the scopolamine injection compared with the vehicle group [approximately 1.6-fold, F (5, 36) = 23.476; P < 0.001]. The GJD treatment remarkably normalized the alterations in AChE activity compared with the control group (P < 0.05 for 100 mg/kg, P < 0.001 for 400 mg/kg; Fig 3A). THA also significantly inhibited the AChE activity in the hippocampus.

### Western blot analysis of the CREB/BDNF/NGF levels in the hippocampus or HT22 cells

The scopolamine injection significantly reduced the level of the phospho-CREB (0.7-fold), BDNF (0.6-fold) and NGF (0.2-fold) proteins in the hippocampus compared with the vehicle group [F (5, 12) = 5.365; P < 0.05 for phospho-CREB, F (5, 12) = 8.653; P < 0.01 for BDNF, F (5, 12) = 13.908; P < 0.01 for NGF], whereas the GJD treatment attenuated those reductions compared with the control group (P < 0.05 or P < 0.01 for both 200 and 400 mg/kg; Fig 3B and 3D). THA only exerted similar effects on the hippocampal level of the NGF protein. From the *in vitro* experiment using the hippocampal neuron, GJD treatment significantly enhanced BDNF level [F (4, 10) = 9.371; P < 0.05 for 0.01, 0.1, 1 μg/mL, P < 0.01 for 10 μg/mL; Fig 3C and 3D].

### Changes in gene expression in the hippocampus

**Fig 4:**
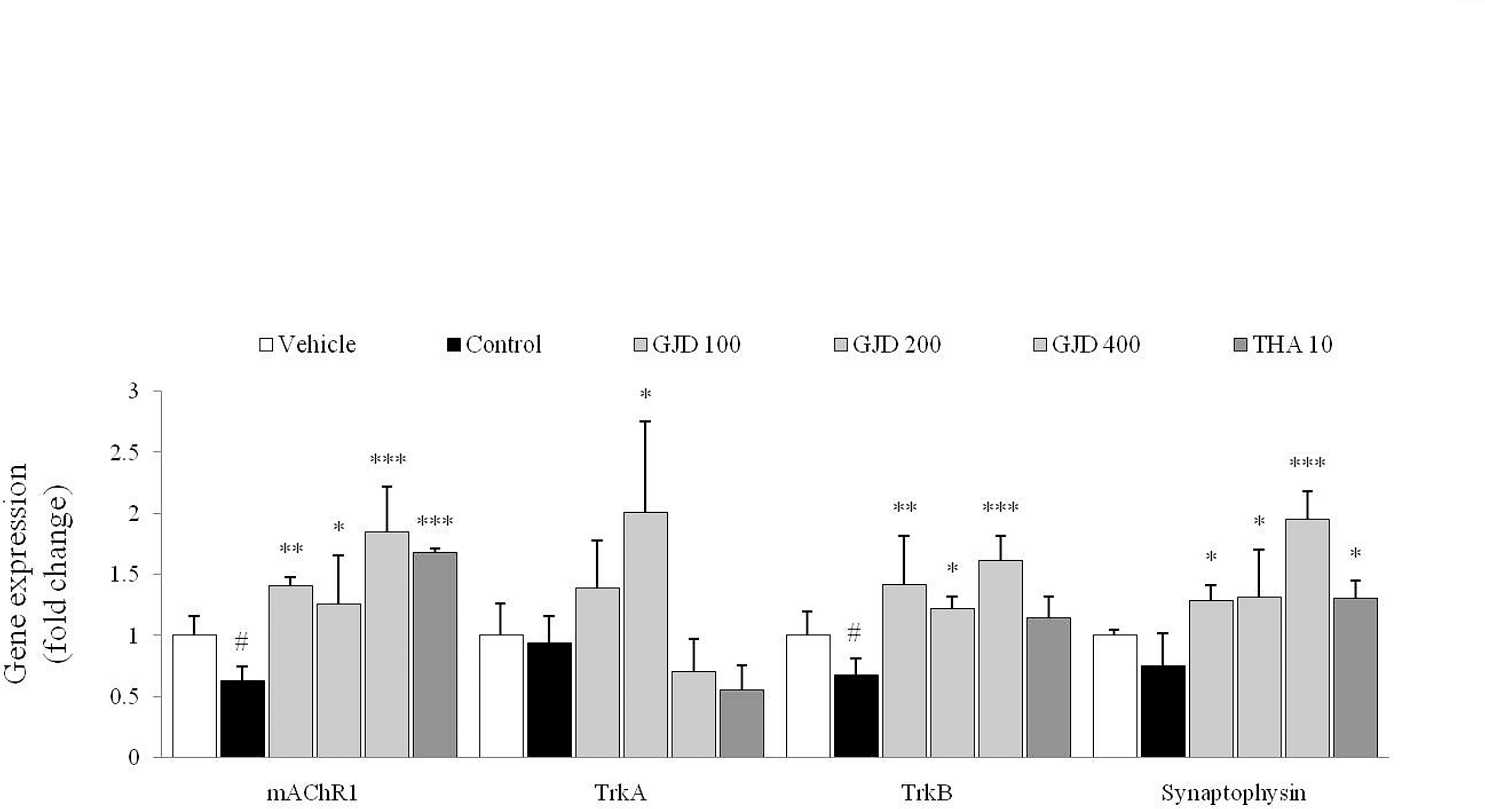
Hippocampal expression levels of the mAChR1, TrkA, TrkB and synaptophysin mRNA. Alterations in the expression levels of the mAChR1, TrkA, TrkB and synaptophysin mRNA were determined by real-time PCR. Gene expression was normalized to that of β-actin. The data are expressed as the means ± SD (*n* = 3). ^#^P < 0.05 compared with the vehicle group; *P < 0.05, **P < 0.01, and ***P < 0.001 compared with the control group.

The scopolamine injection significantly reduced the hippocampal gene expressions of the mAChR1 [F (5, 12) = 13.913; P < 0.05], TrkB [F (5, 12) = 8.407; P < 0.05], TrkA [F (5, 12) = 7.212; P > 0.05] and synaptophysin [F (5, 12) = 11.863; P > 0.05] mRNA compared with the vehicle group. The GJD treatment significantly up-regulated the expression of the mAChR1, TrkA, TrkB and synaptophysin mRNA compared with the control group (P < 0.05 or P < 0.01 or P < 0.001 for 100, 200 and 400 mg/kg, but P < 0.05 for 200 mg/kg in TrkA only; Fig 4). THA had effects similar to those of the GJD treatment on the expression of the mAChR1 and synaptophysin mRNA.

### Immunohistochemistry in the hippocampus

**Fig 5:**
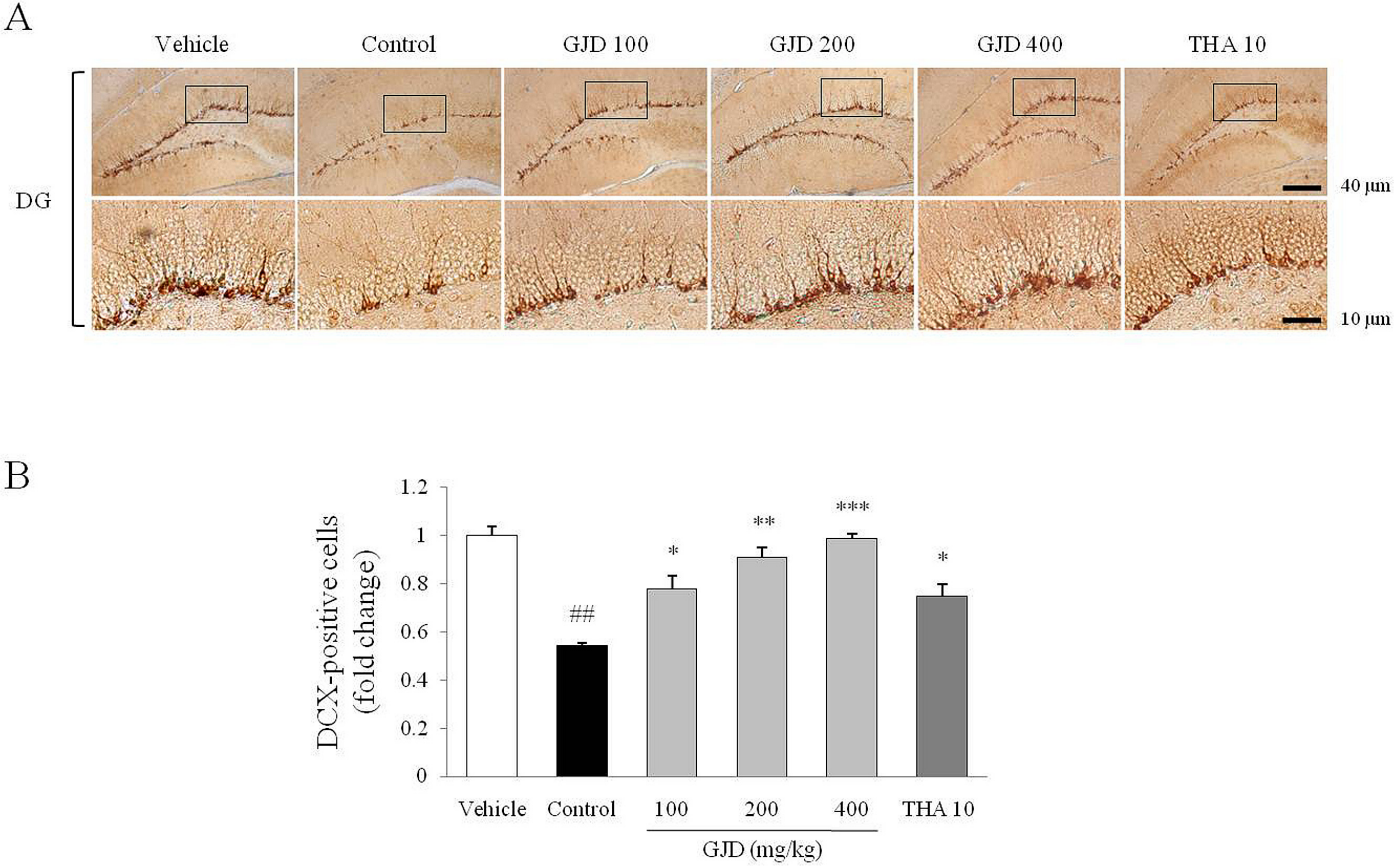
Immunohistochemistry of the DCX-positive cells in the hippocampus. DCX-positive neural progenitor cells were stained in the subgranular zone of the hippocampus. Representative photomicrographs were taken at magnifications of 100 and 400× (A). The intensity of the DCX-positive staining was quantified (B). The data are expressed as the means ± SD (*n* = 3). ^##^P < 0.01 compared with the vehicle group; *P < 0.05, **P < 0.01, and ***P < 0.001 compared with the control group.

DCX staining was used to examine the subgranular zone of the hippocampal DG region and showed that number of neural progenitor cells was significantly reduced following the scopolamine injection compared with the vehicle group [F (5, 12) = 58.014; P < 0.01]. This alteration was significantly attenuated by the GJD treatment compared with the control group (P < 0.05, P < 0.01 and P < 0.001 for 100, 200 and 400 mg/kg, respectively; Fig 5A and 5B). THA has a shown similar effect to GJD on the DCX staining results.

### Histochemistry in the hippocampus

**Fig 6:**
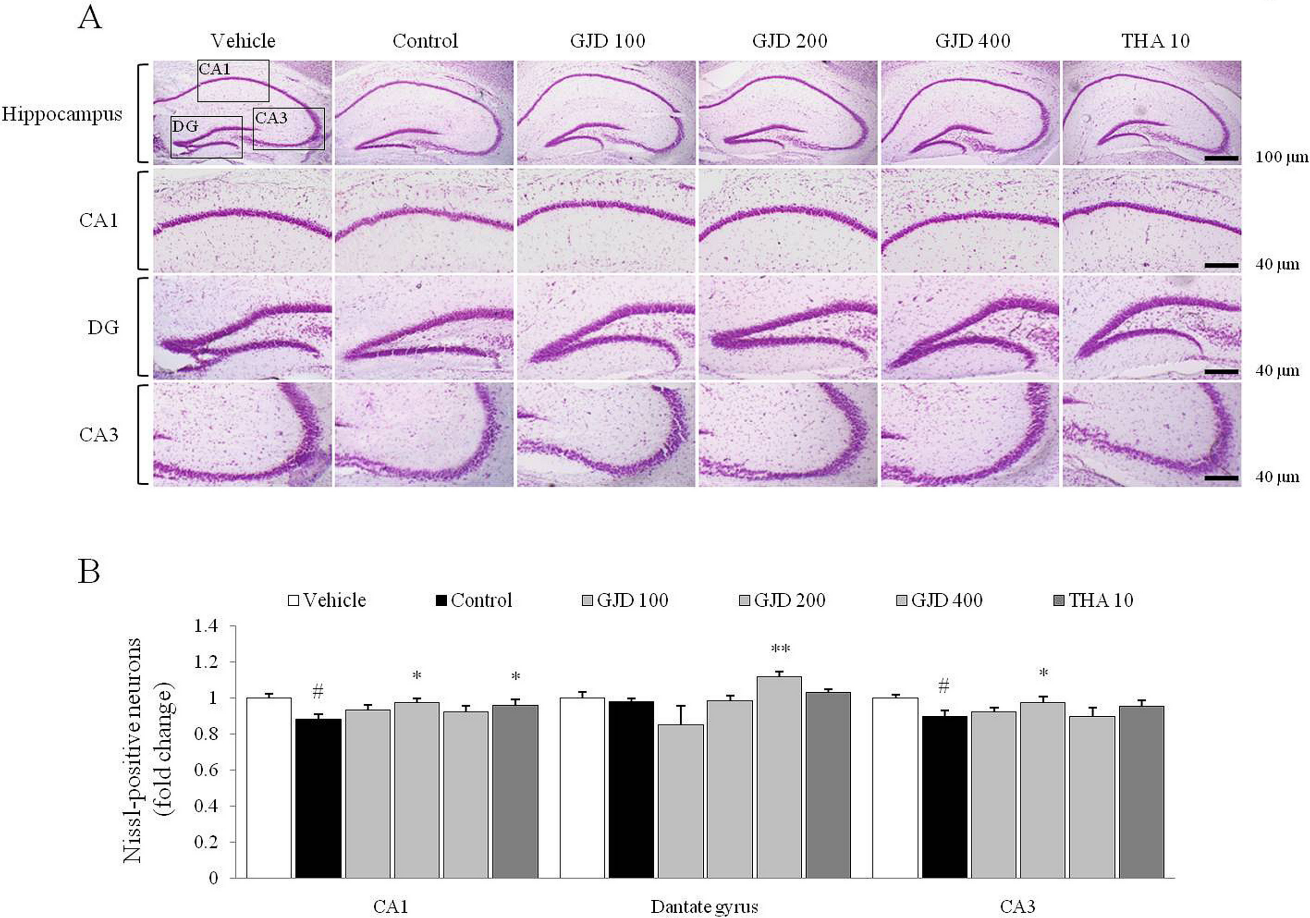
Histochemical findings of the Nissl staining in the hippocampus. The neuronal cell morphology in the hippocampus was detected using Nissl staining. Representative photomicrographs were taken at magnifications of 40 and 100× (A). The intensity of the labeled hippocampal neurons was quantified (B). The data are expressed as the means ± SD (*n* = 3). ^#^P < 0.05 compared with the vehicle group; *P < 0.05, **P < 0.01 compared with the control group.

Nissl staining was used to confirm structural atrophy and showed that the numbers of neuron in the hippocampal CA1 and CA3 regions (but not in the DG region) were significantly reduced following the scopolamine injection compared with the vehicle group [F (5, 12) = 33.724; P < 0.05 and F (5, 12) = 8.922; P < 0.05, respectively], but these alterations were significantly attenuated by the GJD treatment compared with the control group (P < 0.05 or P < 0.01 for 200 or 400 mg/kg; Fig 6A and 6B).

## Discussion

GJD is well-known to maintain body homeostasis and enhance brain activity, and it has been prescribed for patients with central nervous system disorders in Asia. GJD is approved by the MFDS, and annual revenue is estimated to be approximately 46 million USD. Previous studies proved that GJD reduces the infracted tissue volume in a transient middle cerebral artery occlusion model [15], and regulates NGF level in a PC12 cell model [14]. Our previous study also showed that GJD had anti-chronic fatigue properties via regulating muscle and central fatigue-related mechanisms [16].

Based on these reports, we hypothesized that GJD may have an anti-amnesic effect by modulating the cholinergic pathway. To verify our hypothesis, we adopted an animal model of hippocampal memory impairment by injecting scopolamine. Experiments consisted of two parts as follows: experiment 1 (repeated scopolamine injection during training) to test GJD effects on learning process in maze task, and experiment 2 (single scopolamine injection without training) to evaluate GJD effects on biochemical aspects excluding influence by training process. Scopolamine, a competitive muscarinic acetylcholine receptor antagonist, is commonly used to impair memory in rodent models [17]. We injected scopolamine for total 5 times (2 mg/kg per each injection) based on our preliminary study, and used THA, an approved acetylcholinesterase inhibitor, as a positive reference agent [18].

As expected, the 5 serial injections of scopolamine induced amnesic behavior, as evidenced by the both radial arm maze task and the passive avoidance test. Patients with amnesic disorder exhibit behavioral alterations including disorientation, concentration problem and memory deficiencies [19]. The two behavioral tests described above have been used as memory tasks in many animal studies [20]. The radial arm maze task reflects spatial learning and memory and the passive avoidance test assesses fear-motivated memory retrieval in mice [21, 22]. Our results showed that the GJD treatment (particularly 200 and 400 mg/kg) significantly attenuated the amnesic behavior in both tests (Fig 2A–2D).

We examined the effects of GJD on key modulators of the cholinergic system in the hippocampus. Hippocampal cholinergic dysfunction has been observed in neurodegenerative disorders with memory impairment, including Alzheimer’s disease, in both clinical studies [23] and animal experiments [24, 25]. The scopolamine injection interrupts the transmission of acetylcholine into the post-synaptic membrane, which sequentially enhances AChE activity to catalyze the hydrolysis of acetylcholine [26]. As expected, the GJD treatment attenuated the hyperactivation of AChE activity and down-regulated the expression of the mAChR1 mRNA (Figs 3A and 4). The inhibition of AChE activity enhances basal dendritic LTP in the hippocampal CA1 area, and it is mediated by muscarinic M1 receptors [27]. To date, reversible cholinesterase inhibitors have been considered a promising strategy for the pharmacotherapy of mild to moderated Alzheimer’s disease [8].

Acetylcholine signaling eventually derives the phosphorylation of the cAMP response element binding protein (CREB), which then translocates into the nucleus to regulate the transcription of target genes. It is well known that CREB plays a crucial role in neuronal growth, proliferation, differentiation and survival. Numerous studies have also emphasized the interrelationship between the transcriptional activity of CREB and hippocampus-dependent memory formation [28, 29]. In our results, scopolamine significantly reduced the phosphorylation of CREB in the hippocampus, which was almost completely reversed by the GJD treatment (Fig 3B and 3D). BDNF and NGF are two central neurotrophins that modulate memory and participate in the phosphorylation of CREB [30]. A previous study proposed that BDNF could be a diagnostic biomarker in patients with early Alzheimer’s disease and mild cognitive impairment [31]. In the present study, the GJD treatment almost completely restored the levels of the BDNF and NGF proteins, and their receptors (TrkA and TrkB) in the hippocampus, which were considerably decreased by scopolamine (Fig 3B and 3D and Fig 4). GJD also increased the hippocampal expression of the synaptophysin mRNA (Fig 4). Synaptophysin is a major synaptic vesicle protein; it is associated with memory-related behavior [32] and it has been linked to NGF activity in animal studies [33].

It is well known that the hippocampus is a pivotal region of the brain for learning and memory. Adult hippocampal neurogenesis plays an important role in memory formation; therefore, impaired neurogenesis and neuronal integration are regarded as pathological features of neurodegenerative disorders [34]. As expected, the GJD treatment notably reversed the scopolamine-induced inhibition of neurogenesis in the subgranular zone of the hippocampal DG region (Fig 5A and 5B) and alterations in the hippocampal structure in CA1 and CA3 (Fig 6A and 6B). This neurogenesis is known to depend on the activities of both neurotrophins and their receptors [35]. A clinical study presented an association between lower level of BDNF and hippocampal volume shrinkage in older adults [36]. In addition, pyramidal neurons of CA3 were reduced in volume by repeated scopolamine injection in a rat model [37]. The slight shrinkage of hippocampal volume in our result would be attributable to decline of BDNF level. This was partially supported by *in vitro* result, which GJD notably activated BDNF molecule in mouse hippocampal neuronal cells, HT-22 cell line (Fig 3C and 3D). Generally, GJD showed similar activity to THA, suggesting that GJD may have a potent anti-amnesic activity by regulating the cholinergic pathway.

Taken together, this study strongly showed that GJD exerted anti-amnesic actions in a mice model of scopolamine-induced amnesia. The underlying mechanisms involve the regulation of the hippocampal cholinergic system and neurogenesis via activation of BDNF.

## Supporting Information

S1 File Fig 3B and 3C full-length gel and blots. (DOCX)
