# Supplementary material for: Gongjin-Dan Enhances Hippocampal Memory in a Mouse Model of Scopolamine-Induced Amnesia
Source: PLoS One. 2016 Aug 2;11(8):e0159823. doi: 10.1371/journal.pone.0159823 (PMC4970723; doi:10.1371/journal.pone.0159823)
Supplement: S1 File — (DOCX) [file pone.0159823.s001.docx]

***Gongjin-Dan* enhances hippocampal memory in a mouse model of scopolamine-induced amnesia**

Jin-Seok Lee ^a^, Sung-Shin Hong ^b^, Hyeong-Geug Kim ^a^, Hye-Won Lee ^c^, Won-Yong Kim ^a^, Sam-Keun Lee ^d^, and Chang-Gue Son ^a, *^

*^a^ Liver and Immunology Research Center, Oriental Medical Collage of Daejeon University, 22-5 Daehung-dong, Jung-gu, Daejeon, 301-724, Republic of Korea*

*^b^* *Korean Medical College of Daejeon University, 22-5 Yongwoon-dong, Dong-gu, Daejeon301-724, Republic of Korea*

*^c^* *TKM-based Herbal Drug Research Group, Korea Institute of Oriental Medicine, Daejeon 305-811, Republic of Korea*

*^d^* *Department of Applied Chemistry, Daejeon University, 62, Daehak-ro, Dong-gu, Daejeon 34520, Republic of Korea*

**Supplementary information**

**Figure 3B full-length gel and blots**

**
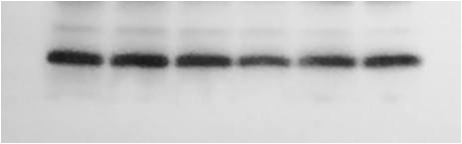
** CREB (37 kDa)

**
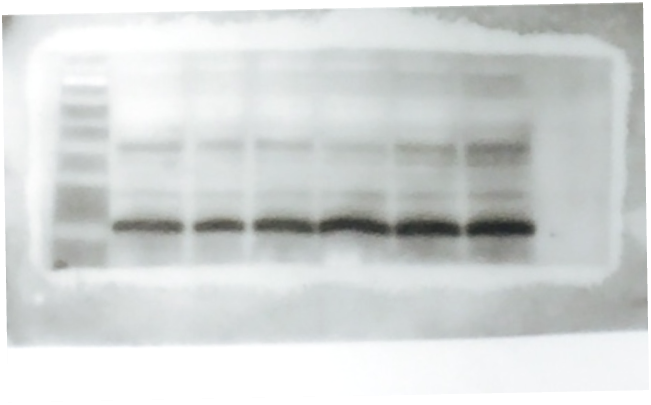
** p-CREB (46 kDa)

**
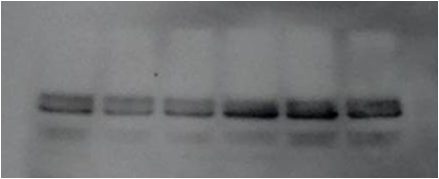
** BDNF (28 kDa)

**
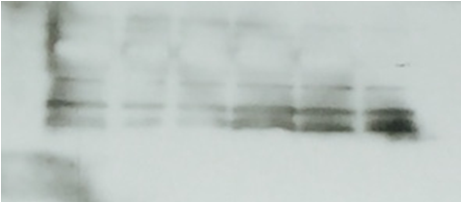
** NGF (30 kDa)

**
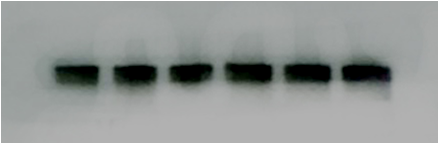
** *β*-actin (44 kDa)

**Figure 3C full-length gel and blots**


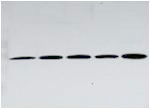
 BDNF (28 kDa)


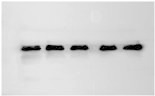
 *β*-actin (44 kDa)
